# Editorial: Deep neural network architectures and reservoir computing

**DOI:** 10.3389/frai.2025.1676744

**Published:** 2025-08-21

**Authors:** Sou Nobukawa, Arya Kumar Bhattacharya, Akira Hirose

**Affiliations:** ^1^Graduate School of Information and Computer Science, Chiba Institute of Technology, Narashino, Japan; ^2^Department of Computer Science, Chiba Institute of Technology, Narashino, Japan; ^3^Research Centre for Mathematical Engineering, Chiba Institute of Technology, Narashino, Japan; ^4^Department of Preventive Intervention for Psychiatric Disorders, National Institute of Medicine Mental Health, National Center of Neurology and Psychiatry, Tokyo, Japan; ^5^Research and Development, Mahindra University, Hyderabad, India; ^6^Department of Computer Science and Artificial Intelligence, Mahindra University, Hyderabad, India; ^7^Department of Electrical Engineering and Information Systems, The University of Tokyo, Tokyo, Japan; ^8^Department of Bioengineering, The University of Tokyo, Tokyo, Japan

**Keywords:** deep echo state network, deep learning, dynamics, echo state network, reservoir computing

## 1 Introduction

Over the past decade, deep learning (DL) techniques such as convolutional neural networks (CNNs) and long short-term memory (LSTM) networks have played a pivotal role in advancing the field of computational intelligence (Bengio et al., 2021). Recent developments in deep neural network (DNN) architectures and computational infrastructure (particularly parallel computing) have further accelerated progress by supporting the computational demands of optimizing large numbers of network parameters. These advancements have expanded the applicability of DL to a broad range of tasks in computational intelligence (Sharifani et al., 2023).

Simultaneously, reservoir computing (RC) has attracted increasing attention (Tanaka et al., 2019). Typically, RC consists of a fixed recurrent neural network (the reservoir) and a trainable readout layer. It exploits the non-linear spatiotemporal dynamics of the reservoir to transform inputs, while learning is applied only to the output layer. This structure dramatically reduces the number of trainable parameters, resulting in high learning efficiency. However, conventional RC, which typically involves a single reservoir layer, has generally not matched the performance of deeper neural architectures used in mainstream DL.

To overcome this limitation, recent research has proposed deep RC architectures composed of multiple sub-reservoirs arranged in parallel, ring, hub, or multilayer configurations (Iinuma et al., 2022; Kawai et al., 2023; Yan et al., 2024). These approaches aim to enhance the expressive power of the RC while retaining its efficiency. Such advances suggest that deep RC might become a competitive alternative to conventional DL models, opening new avenues for applications in computational intelligence. This Research Topic highlights the recent progress at the intersection of DL and RC. These contributing articles explore novel architectures and emerging applications, reflecting the current trends in both DNNs and RC.

## 2 Deep neural network architectures

In this Research Topic, three articles address practical identification problems through the proposal of novel DL network architectures. The first article by Chincholi and Koestler explores a promising transformer-based approach for glaucoma detection from retinal images. Their study employs two transformer architectures—Vision Transformer and Detection Transformer—to identify and analyze crucial anatomical features (e.g., optic disc and optic cup). By computing the cup-to-disc ratio, a critical glaucoma indicator, their method demonstrates high accuracy, highlighting the potential of transformer models for medical imaging and diagnostic decision support. This innovative research illustrates the effectiveness and interpretability of transformer architectures in healthcare applications. The second article by Al-Mnayyis et al. introduces multi-fusion preprocessing techniques alongside a residual depth-wise network. Using a novel mammography dataset from King Abdullah University Hospital, their proposed architecture outperforms traditional DL models such as MobileNetV2, VGG16, and ResNet50 in breast cancer classification. This contribution underscores the importance of carefully designed preprocessing steps and tailored network architectures for achieving superior diagnostic performance. The third article by Gavali and Banu presents a novel multimodal fusion approach for Indian bird species identification that combines visual and acoustic data. Their work leverages CNNs for image analysis and LSTM networks for acoustic analysis of bird calls. By integrating visual and acoustic features through early and late fusion techniques, their methodology enhances classification accuracy compared to single-modality approaches. This study demonstrates the effectiveness of multimodal fusion strategies in biodiversity monitoring and ecological conservation. Collectively, these three articles, which focus on the practical application of DNNs, are expected to substantially contribute to the advancement of DL research and its practical implementation in various societal contexts.

## 3 Multi-reservoir architectures

This Research Topic features studies involving two types of multi-reservoir approaches. The first is the multi-layered ESN architecture, commonly referred to as deep ESNs. The second is a hybrid reservoir architecture that incorporates the slow and fast dynamic components. In the first approach, Inoue et al. demonstrates that deep ESNs can achieve enhanced performance by propagating signals through multiple reservoir layers, each with its own optimally tuned neuronal leak rate. This layer-wise adjustment allows the network to capture dynamic features across multiple timescales, contributing to the improved representational capacity of deep ESNs. In the second approach, Tokuda and Katori proposed a novel reservoir architecture composed of two types of ESNs: one with slow temporal dynamics for encoding internal system parameter variations, and the other with fast temporal dynamics for capturing system state transitions. This dual-timescale design is promising for modeling systems with bifurcation characteristics, and may represent a key direction in the evolution of RC for dynamical system estimation. Together, these studies provide valuable insights into architectural innovations to improve the performance and interpretability of RC frameworks.

## 4 Conclusions

This Research Topic brings together innovative contributions at the intersection of DL and RC, highlighting the advancement in neural network architectures, computational techniques, and practical applications. In addition to proposing novel architectures for DNNs and advanced RC, the featured articles demonstrate how these methods could address challenges ranging from benchmark dynamic system modeling to real-world applications in domains such as medical diagnostics and ecological conservation. Collectively, these studies underscore the growing importance and potential of interdisciplinary approaches, which are expected to drive future research, technological advancement, and societal impact in the field of computational intelligence.
